# Supplementary material for: Orthotopic Bone Formation by Streamlined Engineering and Devitalization of Human Hypertrophic Cartilage
Source: Int J Mol Sci. 2020 Sep 30;21(19):7233. doi: 10.3390/ijms21197233 (PMC7582540; doi:10.3390/ijms21197233)
Supplement: Supplementary file 1 [file ijms-21-07233-s001.pdf]

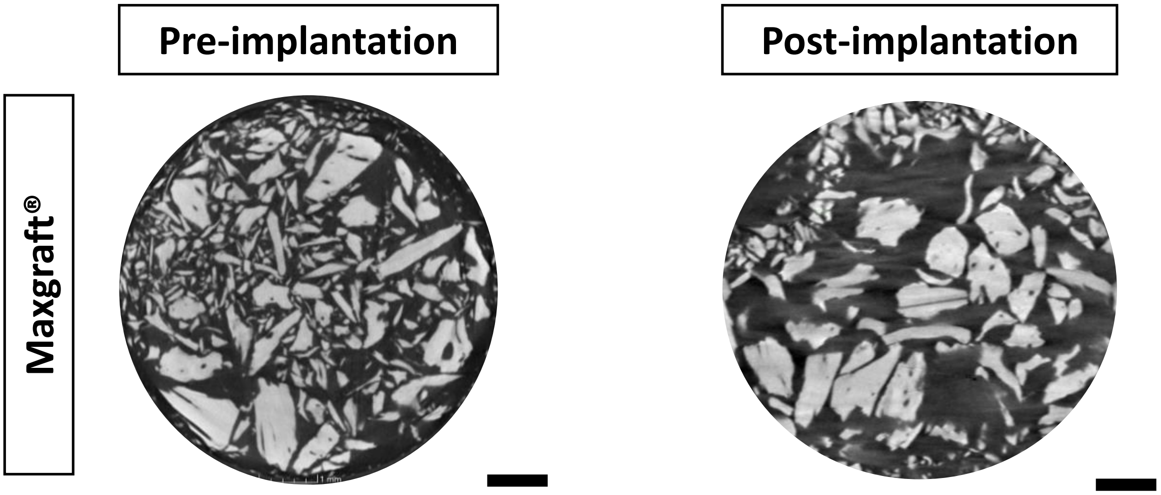

Figure S1: *Implanted material Maxgraft®. Representative uCT picture of the Maxgraft® granules pre- and post- implantation. (scale bar = 1mm).*

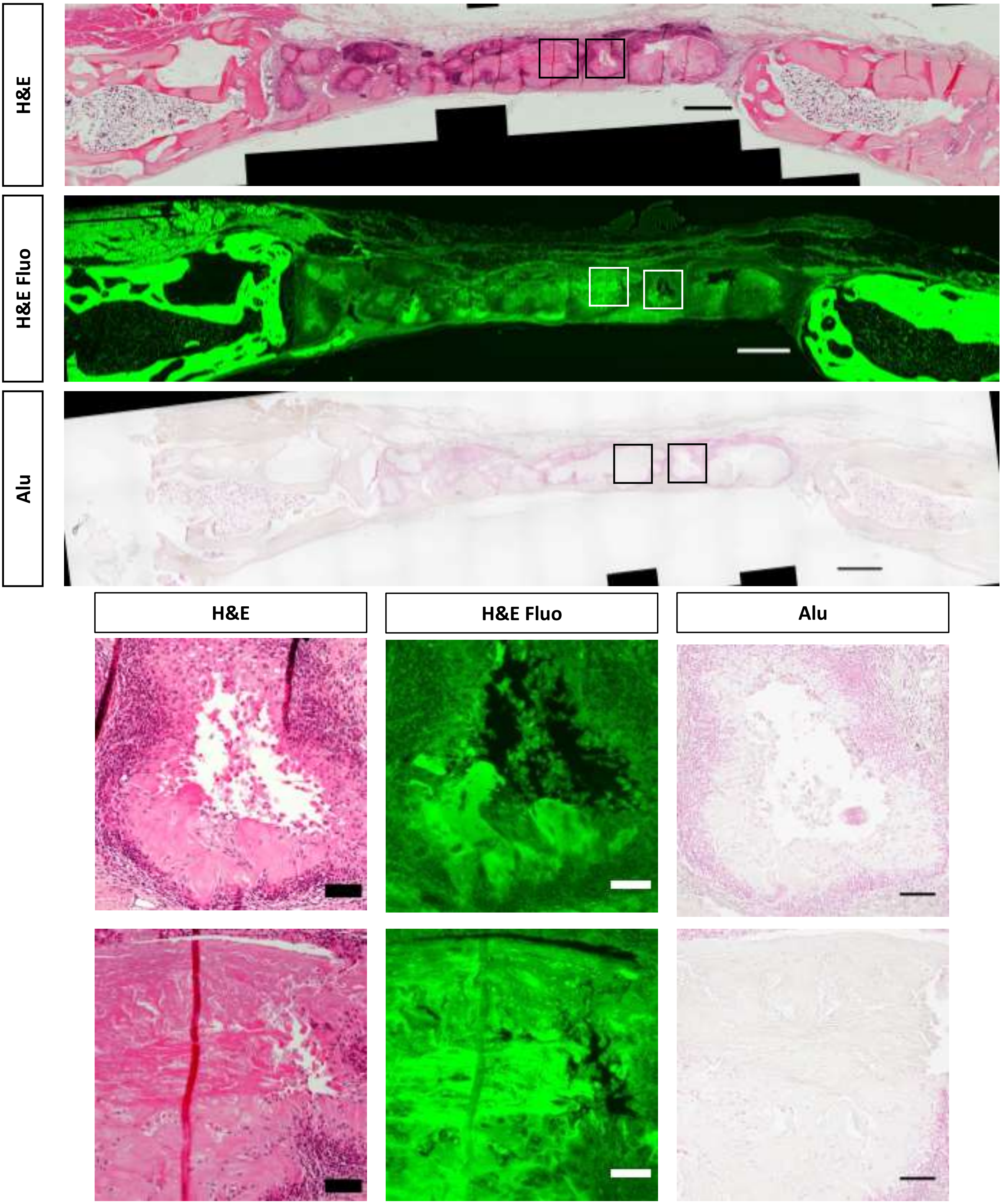

Figure S2: *Histological analysis of ECM implanted rabbit calvarial defect. H&E stains bone in deep pink and shows the autofluorescence of the collagen from the eosin staining. Alu staining stains positively (purple) by in-situ hybridization for human specific Alu sequences. (scale bar = 1-mm for whole section and 100-µm for close up).*
